# Association of Dietary Flavonoids Intake With All-Cause and Cardiovascular Disease Mortality in Diabetic Kidney Disease: A Cohort Study From the NHANES Database

**DOI:** 10.1155/2024/8359294

**Published:** 2024-11-04

**Authors:** Qian Wang, Weizhu Deng, Jian Yang, Yaqing Li, Hui Huang, Yayong Luo, Zhongxia Li, Zheyi Dong

**Affiliations:** ^1^Department of Nephrology, First Medical Center of Chinese PLA General Hospital, Nephrology Institute of the Chinese People's Liberation Army, National Key Laboratory of Kidney Diseases, National Clinical Research Center for Kidney Diseases, Beijing Key Laboratory of Kidney Disease Research, Beijing 100853, China; ^2^Shenzhen Traditional Chinese Medicine Hospital, Shenzhen District, Guangdong 518033, China; ^3^BYHEALTH Institute of Nutrition & Health, Guangzhou District 510663, China

**Keywords:** all-cause mortality, cardiovascular disease mortality, diabetic kidney disease, flavonoid

## Abstract

The relationship between dietary flavonoid intake and mortality in the diabetic kidney disease (DKD) population is unknown. So this study is aimed at investigating the association of total dietary flavonoid intake and their subclasses with all-cause and cardiovascular disease (CVD) mortality. Data of this cohort study were extracted from the NHANES (2007–2010 and 2017–2018). The survival status of participants was determined by linking to the National Death Index through the end of 2019. Flavonoid intake was measured using two 24-h dietary recall interviews. The Kaplan–Meier curves and weighted Cox proportional hazard regression models were used to assess the effect of dietary flavonoid intake on CVD and all-cause mortality, with adjustments for multiple covariates. A total of 1155 participants were included for analysis. After a median follow-up of 76.36 (S.E: 3.24) months, 409 participants died of all-cause mortality, of which 138 died of CVD. In the fully adjusted model, higher total dietary flavonoids intake (HR = 0.69, 95% CI: 0.52–0.92) was associated with lower all-cause mortality and subclasses of higher flavones (HR = 0.60, 95% CI: 0.35–0.85) was also with lower all-cause mortality. In subclasses of flavonoids, higher intake of both anthocyanidins (HR = 0.54, 95% CI: 0.28 to 0.87) and flavones (HR = 0.50, 95% CI: 0.28–0.87) were associated with lower odds of CVD mortality. Higher flavonoid intake was associated with a reduced risk of CVD and all-cause mortality in DKD. Higher flavonoid intake provides a potential opportunity to improve the prognosis of DKD. And future research into the mechanisms between flavonoids and mortality is needed.

## 1. Introduction

Diabetic kidney disease (DKD) is a common microvascular complication of diabetes mellitus (DM), characterized by a declining glomerular filtration rate (GFR), persistent proteinuria, and structural kidney alterations, ultimately leading to renal failure [1]. DKD is associated with increased mortality risk [2, 3]. The 10-year cumulative all-cause mortality rate for individuals with DM and chronic kidney disease (CKD) is estimated at 31.1%, compared to 11.5% for those with diabetes but without CKD, and 7.7% for those without either condition [4]. DKD presents a threefold higher risk of all-cause mortality and a 16-year reduction in life expectancy compared to the general population [5]. Despite the effectiveness of current DKD management strategies, mortality among individuals with DKD remains strikingly high [6].

Dietary modifications can potentially improve the prognosis of DKD [3]. High consumption of fruits and vegetables is linked to a reduced risk of DKD, with flavonoids in these foods likely contributing to this effect [7, 8]. Flavonoids are natural compounds in fruits, vegetables, tea, and wine [9, 10]. Emerging research indicates that dietary flavonoid intake plays a crucial role in the progression and prevention of various diseases, including metabolic disease [11], cardiovascular disease (CVD) [12], and inflammatory disease [13]. A recent study showed that a higher dietary flavonoid intake, particularly flavan-3-ols, flavones, and anthocyanidins, is associated with a lower risk of developing DKD [14]. Antioxidant and anti-inflammatory properties are potential mechanisms through which flavonoids may mitigate DKD [15, 16]. The pathogenesis of DKD is related to inflammation and oxidative stress [17]. In addition, flavonoids may hold promise in mitigating CVD complications in DM patients by improving CVD risk factors [18]. Anthocyanidins, in particular, can influence CVD risk through various mechanisms, including improving lipid profiles and endothelial function [19]. Population-based studies have demonstrated an association between flavonoid intake and reduced risk of all-cause and CVD mortality [20, 21]. However, studies on flavonoid intake and long-term outcomes in DM patients are still limited, and the impact on mortality risk in DKD patients remains unreported.

This cohort study is aimed at investigating the relationship between flavonoid intake, different subclasses of flavonoids, and mortality risk (both all-cause mortality and CVD mortality) in patients with DKD.

## 2. Methods

### 2.1. Study Design and Participants

The National Health and Nutrition Examination Survey (NHANES), carried out by the National Center for Health Statistics (NCHS) of the U.S. Centers for Disease Control and Prevention, is a comprehensive, ongoing, and nationally representative health survey. In this cohort study, data on DKD were extracted from the database (2007–2008, 2009–2010, and 2017–2018). Interviews and medical examinations were performed to gather demographic, socioeconomic, dietary, physiological, and laboratory data. The survey protocol was approved by the National Center for Health Statistics Research Ethics Review Board. All participants provided their written informed consent. The current study was exempt from further review by the hospital.

Individuals aged 20 years or older with DKD were included in the analysis. Participants who had diabetes and met the criteria for CKD were defined as DKD [22]. DM was defined as meeting any of the subsequent criteria: (1) HbA1c ≥ 6.5%; (2) a 2-h 75 g oral glucose tolerance test (OGTT) ≥ 200 mg/dL, fasting glucose ≥ 126 mg/dL; (3) self-reported DM which diagnosis by a physician; and (4) utilization of insulin or other diabetes medication [23]. CKD was defined as the urinary albumin-to-creatinine ratio (UACR) ≥ 30 mg/g and/or estimated glomerular filtration rate (eGFR) < 60 mL/min/1.73 m^2^ [24]. Participants with missing information about dietary intake and survival were excluded.

### 2.2. Dietary Flavonoid Intake Assessment

Data on total flavonoid intake were obtained from the NHANES and the Food and Nutrient Database for Dietary Studies (FNDDS) databases. This study focused on six flavonoid subclasses: flavones, flavan-3-ols, anthocyanidins, flavanones, flavonols, and isoflavones. Dietary intake data were collected by trained dietitians through two 24-h food recall interviews. The first interview was conducted at the mobile examination centers, while the second interview was conducted by telephone after 3–10 days. Detailed information on the calculation of dietary flavonoid intake is available at https://www.ars.usda.gov/northeast-area/beltsville-md-bhnrc/beltsville-human-nutrition-research-center/food-surveys-research-group/docs/fndds/.

### 2.3. Covariates

Potential covariates included age, race, educational level, marriage, physical activity, overweight, diabetic retinopathy, hypertension, CVD, uric acid, lymphocyte, platelet, hemoglobin, calcium intake, potassium intake, phosphorus intake, and dialysis. Demographic and lifestyle information, such as age, race, educational level, marriage status, and physical activity, was gathered through a household interview. Participants' height, weight, and body mass index (BMI) were measured at a Mobile Examination Center. Information on calcium, potassium, and phosphorus intake was collected through two 24-h dietary interviews. Diabetic retinopathy was defined as a “yes” response to the question, “Diabetes affected eyes/had retinopathy” [25]. Hypertension was defined as either taking antihypertensive medications, having a systolic blood pressure ≥ 130 mmHg and/or diastolic blood pressure ≥ 80 mmHg, or having been diagnosed by a physician [26]. CVD was defined as a diagnosis by a physician of conditions such as angina, heart failure, coronary heart disease, stroke, congestive heart failure, or the use of cardiovascular medications [27]. Dialysis was defined as a positive response to the question “Have you received dialysis in the past 12 months?” [28].

### 2.4. Outcome and Follow-Up

The National Death Index (NDI) is the centralized database of the NCHS that records all deaths in the United States [29]. Deaths were obtained through NDI to December 31, 2019. All-cause mortality and CVD mortality were included in the outcomes. All-cause mortality was defined as any reason for death. CVD mortality was defined as deaths from heart disease (I00-I09, I11, I13, and I20-I51) based on the International Classification of Disease, 10th Revision. Follow-up of each participant was calculated from the date of recruitment to the date of death or the end of follow-up (December 31, 2019), whichever occurred first. All data from this study were publicly available (https://www.cdc.gov/nchs/datalinkage/mortality-public.htm).

### 2.5. Statistical Analysis

Masked variance in the primary sampling unit, pseudo strata, and sampling weights were utilized to account for the multistage sampling design and ensure nationally representative estimates. Categorical variables were reported as frequencies and percentages (%), while continuous variables were expressed as means and standard errors (S.E). Chi-square tests and weighted *t*-tests were performed to compare categorical and continuous variables across different groups. Confounders were identified based on significant associations with outcomes in the weighted univariate Cox proportional hazard model. Kaplan-Meier (KM) curves as well as weighted univariate and multivariate Cox proportional hazard models were used to investigate the relationship between flavonoids and all-cause mortality or CVD mortality. Mortality risk was described using a hazard ratio (HR) and 95% confidence interval (CI). The associations were further explored in different smoking status and BMI subgroups. Missing values were imputed using random forest-based multiple imputation by chained equations. *p* < 0.05 was defined as statistically significant. Processing of missing values was performed using Python 3.9, and all statistical analyses were performed with SAS 9.4 (SAS Institute Inc., Cary, NC, United States).

## 3. Results

### 3.1. Characteristics of Participants

**Figure 1:**
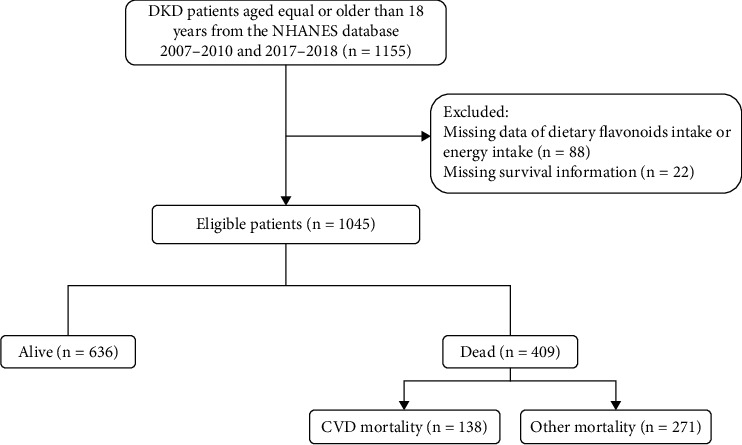
Flow chart of participant selection.

A total of 1155 DKD patients were initially included in the database. Then, participants were excluded from those with dietary intake missing (*n* = 88) and survival information missing (*n* = 22). Finally, 1045 participants were recorded from the NHANES.  Figure 1 shows the screening procedure. There was no statistical difference between missing data after imputation (Table S1). The characteristics of the participants are summarized in Table 1. The mean age of participants was 63.84 years, and 558 (51.53%) were male. During the 76.36-month follow-up period, 409 all-cause mortalities were documented, and 138 were CVD mortality. In the death group, individuals were observed to be older, have lower levels of education, smoking, have a higher BMI, and have hypertension or CVD. Table S2 shows the information on flavonoid intake. The mean daily total dietary flavonoid intake was 114.23 mg (13.26). The main dietary intake subclass of flavonoids was flavan-3-ols.

### 3.2. The Association Between Total Flavonoids and Six Subclasses Intake and All-Cause and CVD Mortality

**Figure 2:**
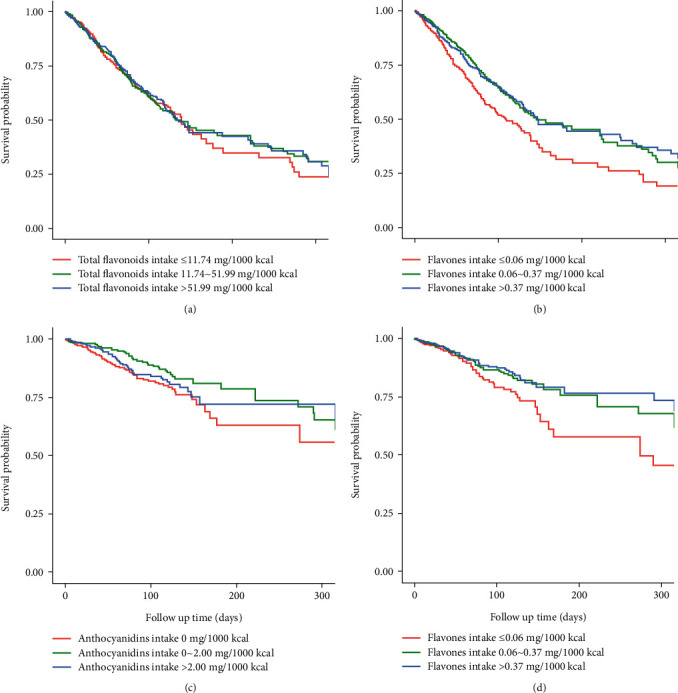
Associations between flavonoid intake with all-cause/CVD mortality. (a) Flavonoid intake and all-cause mortality, (b) flavones and all-cause mortality, (c) anthocyanidins and CVD mortality, and (d) flavones and CVD mortality.

Table 2 shows the relationship between total dietary flavonoids and the six subcategories intake (isoflavones, anthocyanidins, flavan-3-ols, flavanones, flavonols, and flavones) with all-cause and CVD mortality. In the adjusted model of all-cause mortality, higher intake of total flavonoids (HR = 0.69, 95% CI: 0.52–0.92) and flavones (HR = 0.60, 95% CI: 0.35–0.85) were associated with lower odds of all-cause mortality. The KM curves showed significantly lower all-cause mortality in patients with higher flavonoids and flavone intake compared to those with lower intake levels (Figures 2(a) and 2(b)). In the adjusted model of CVD mortality, higher intake of anthocyanidins (HR = 0.54, 95% CI: 0.28–0.87) and flavones (HR = 0.50, 95% CI: 0.28–0.87) were also associated with lower odds of CVD mortality. The KM survival curves also showed that higher anthocyanidins and flavones intake were associated with lower CVD mortality (Figures 2(c) and 2(d)).

### 3.3. The Association Between Subclasses of Flavones With All-Cause Mortality

The relationship between the subclass of flavones and all-cause mortality was further explored.  Table 3 illustrates the association between flavones with all-cause mortality. In flavones, higher intake of apigenin (HR = 0.67, 95% CI: 0.50–0.90) and luteolin (HR = 0.67, 95% CI: 0.52–0.87) were also associated with all-cause mortality.

### 3.4. The Association Between Subclasses of Anthocyanidins and Flavones With CVD Mortality

The association between subclasses of flavonoids (anthocyanidins and flavones) and cardiovascular mortality was also investigated, and the findings are summarized in Table 4. In anthocyanidins, a higher intake of pelargonidin (HR = 0.58, 95% CI: 0.35–0.95) was associated with lower odds of CVD mortality. In flavones, a higher intake of luteolin (HR = 0.52, 95% CI: 0.31–0.88) was associated with lower odds of CVD mortality.

### 3.5. The Association of Flavonoid Intake With All-Cause and CVD Mortality in Different Subgroups

We further investigated the relationship of flavonoid intake with all-cause and CVD mortality in different smoking status and BMI subgroups (Tables 5 and 6). Higher flavone intake was related to lower all-cause mortality risk in DKD patients with different smoking statuses and BMI. Apigenin intake > 0.04 was linked to decreased all-cause mortality risk in DKD patients with smoking (HR = 0.63, 95% CI: 0.42–0.96) and BMI ≥ 25 kg/m^2^ (HR = 0.63, 95% CI: 0.44–0.89). Increased luteolin intake was linked to lower all-cause mortality risk in DKD patients without smoking (HR = 0.60, 95% CI: 0.37–0.97) and different BMI. Flavone intake > 0.37 was related to decreased CVD mortality risk in DKD patients with smoking (HR = 0.40, 95% CI: 0.21–0.75) and BMI ≥ 25 kg/m^2^ (HR = 0.47, 95% CI: 0.25–0.88). Similar findings were observed between elevated luteolin intake and lower CVD mortality in patients with smoking status (HR = 0.44, 95% CI: 0.22–0.89) and BMI ≥ 25 kg/m^2^ (HR = 0.46, 95% CI: 0.26–0.83).

## 4. Discussion

We assessed the association between total flavonoids and their subclass intake and all-cause and CVD mortality in patients with DKD. The study suggested that intake of higher flavonoids may associated with lower odds of mortality in DKD. Higher flavone intake was associated with lower odds of all-cause mortality, whereas higher anthocyanidins and flavone intake were associated with lower CVD mortality.

Higher intake of flavones was related to lower odds of all-cause mortality. Flavonoids have been shown to vasodilate and improve vascular endothelial function by promoting nitric oxide production and angiotensin-converting enzyme inhibition [30, 31]. These actions may help to maintain proper vascular health and integrity, thereby reducing the risk of death. Higher intakes of flavones were consistently associated with lower risks of death from all CVDs, ischemic heart disease, cerebrovascular disease, and peripheral artery disease [20]. Flavonoids have been shown to possess antioxidant and anti-inflammatory properties [9, 32]. These compounds are potent antioxidants, capable of scavenging free radicals and reducing oxidative stress [32]. In DKD, increased oxidative stress and inflammation play a crucial role in the progression of renal damage and cardiovascular complications [17, 33, 34]. By reducing oxidative stress and inflammation, flavones may help protect against cellular damage and preserve renal and cardiovascular function.

A higher intake of anthocyanidins and flavones resulted in lower odds of CVD mortality. This finding is in line with previous research that has suggested a cardioprotective role for these subclasses of flavonoids [20, 35]. Ponzo et al. [36] reported that anthocyanidins were inversely correlated with incident cardiovascular events. Furthermore, the study showed that dietary flavonoids improve vascular endothelial cell function. Anthocyanidins are water-soluble flavonoids derived mainly from colored fruits, vegetables, and flowers [37]. Some studies showed a reduction in arterial stiffness, endothelial function, and serum lipids [9]. Anthocyanidins also have been shown to improve glucose metabolism and insulin sensitivity, which is critical for controlling blood sugar in diabetics [37]. Flavonoids were found to lower blood glucose and improve insulin signaling pathways by increasing insulin secretion in animal models [38]. Better control of blood glucose reduces diabetes-related complications, which in turn reduces mortality.

Our study suggested that higher flavones and luteolin intake were associated with decreased CVD mortality specifically in patients with smoking and those with BMI ≥ 25 kg/m^2^. In individuals with smoking behaviors, flavones may exert protective effects through their potent antioxidant and anti-inflammatory properties. In smokers, oxidative stress and chronic inflammation are exacerbated, contributing to endothelial dysfunction and increased cardiovascular risk [39]. Flavones, including luteolin, have been shown to mitigate oxidative stress and inflammation by scavenging free radicals and modulating inflammatory pathways [40, 41]. Their high antioxidant capacity could, therefore, counteract the oxidative damage induced by smoking, potentially explaining the observed association with reduced CVD mortality. Similarly, for individuals with BMI ≥ 25 kg/m^2^, which indicates overweight or obesity, the impact of flavonoids might be linked to their role in modulating metabolic and inflammatory processes. Obesity is characterized by chronic low-grade inflammation and increased oxidative stress, which contributes to cardiovascular risk [42]. Flavones and luteolin have been documented to improve metabolic profiles and reduce inflammation, potentially offering cardiovascular protection [43].

Promoting a higher intake of flavonoid-rich foods among patients with DKD could have significant implications for their long-term health outcomes. To fully capitalize on this potential, it is critical to address two concerns. First, it is essential to increase the medical staff's awareness of the beneficial effects of flavonoids in DKD. By disseminating this knowledge to physicians and other healthcare providers, a timely intervention and individualized treatment plan can be ensured which emphasizes the importance of flavonoid intake as part of a patient's comprehensive care. Second, timely interventions are needed to encourage patients to consume more flavonoids in their diets. By facilitating dietary modifications and providing education about flavonoid sources, healthcare professionals can allow patients to make wise choices when choosing foods.

Strengths of this study included substantial sample size and comprehensive assessment of the relationship between total flavonoid and six subclasses intake with mortality. This study still has several limitations. Firstly, dietary intake information was determined based on a 24-h recall interview, which may be subject to self-report bias. Secondly, flavonoid intake was only assessed at baseline, which meant that it was not possible to capture changes in dietary patterns over the follow-up period. Lastly, the results only represent the US population, and the findings cannot be directly extrapolated to the general population worldwide.

In the current study, we found a beneficial relationship between the dietary flavonoid intake and the risk of mortality in DKD patients. Specifically, a higher intake of flavones was associated with lower odds of all-cause mortality, while a higher intake of anthocyanidins and flavones was also related to lower odds of CVD mortality. Future studies are needed to elucidate the specific pathways between flavonoid intake and mortality in patients with DKD, and interventional studies are needed to confirm our findings.

## Figures and Tables

**Table 1:** Characteristics of DKD patients.

**Variables**	**Total (** **n** = 1045**)**	**All-cause mortality**				**CVD mortality**			
		**No (** **n** = 636**)**	**Yes (** **n** = 409**)**	**Statistics**	**p**	**No (** **n** = 907**)**	**Yes (** **n** = 138**)**	**Statistics**	**p**
Age, years, mean (S.E)	63.84 (0.65)	61.12 (0.89)	68.84 (0.80)	*t* = −6.42	<0.001	63.32 (0.70)	67.50 (1.12)	*t* = −3.24	0.002
Gender, *n* (%)				*χ* ^2^ = 0.05	0.829			*χ* ^2^ =0.29	0.592
Male	558 (51.53)	337 (51.86)	221 (50.92)			483 (51.23)	75 (53.66)		
Female	487 (48.47)	299 (48.14)	188 (49.08)			424 (48.77)	63 (46.34)		
Race, *n* (%)				*χ* ^2^ = 11.57	0.003			*χ* ^2^ = 9.99	0.007
Non-Hispanic White	403 (60.12)	204 (56.79)	199 (66.24)			332 (58.74)	71 (69.93)		
Non-Hispanic Black	250 (14.72)	152 (14.51)	98 (15.11)			215 (14.48)	35 (16.42)		
Others	392 (25.16)	280 (28.69)	112 (18.65)			360 (26.78)	32 (13.65)		
Education level, *n* (%)				*χ* ^2^ = 35.12	<0.001			*χ* ^2^ = 4.56	0.102
Below high school	423 (29.40)	232 (23.16)	191 (40.88)			359 (28.07)	64 (38.83)		
High school	236 (29.47)	141 (31.49)	95 (25.76)			206 (29.78)	30 (27.24)		
College graduate or above	386 (41.13)	263 (45.36)	123 (33.36)			342 (42.15)	44 (33.93)		
Marriage, *n* (%)				*χ* ^2^ = 8.82	0.012			*χ* ^2^ = 2.13	0.345
Married	526 (53.64)	340 (57.12)	186 (47.24)			461 (54.22)	65 (49.52)		
Never married	87 (7.30)	60 (7.87)	27 (6.26)			76 (7.51)	11 (5.82)		
Others	432 (39.06)	236 (35.01)	196 (46.51)			370 (38.27)	62 (44.66)		
Poverty-to-income ratio, *n* (%)				*χ* ^2^ = 4.91	0.086			*χ* ^2^ = 4.54	0.104
< 1.0	203 (13.90)	118 (12.93)	85 (15.68)			170 (13.41)	33 (17.38)		
≥ 1.0	734 (78.29)	455 (80.38)	279 (74.44)			648 (79.27)	86 (71.34)		
Unknown	108 (7.81)	63 (6.69)	45 (9.88)			89 (7.33)	19 (11.28)		
Smoking, *n* (%)				*χ* ^2^ = 2.69	0.101			*χ* ^2^ = 1.18	0.278
No	497 (47.36)	327 (49.54)	170 (43.35)			439 (48.18)	58 (41.56)		
Yes	548 (52.64)	309 (50.46)	239 (56.65)			468 (51.82)	80 (58.44)		
Drinking, *n* (%)				*χ* ^2^ = 8.35	0.015			*χ* ^2^ = 4.75	0.093
No	396 (36.50)	230 (33.10)	166 (42.76)			346 (36.42)	50 (37.07)		
Yes	549 (53.85)	336 (55.45)	213 (50.90)			470 (53.19)	79 (58.55)		
Unknown	100 (9.65)	70 (11.45)	30 (6.33)			91 (10.39)	9 (4.38)		
Physical activity, *n* (%)				*χ* ^2^ = 45.47	<0.001			*χ* ^2^ = 7.29	0.026
< 450 met × minutes/week	111 (10.54)	72 (11.34)	39 (9.06)			96 (10.49)	15 (10.87)		
≥ 450 met × minutes/week	417 (41.79)	305 (50.14)	112 (26.43)			381 (43.64)	36 (28.71)		
Unknown	517 (47.67)	259 (38.51)	258 (64.51)			430 (45.87)	87 (60.43)		
Diabetic retinopathy, *n* (%)				*χ* ^2^ = 9.65	0.008			*χ* ^2^ = 1.50	0.473
No	576 (56.26)	368 (59.51)	208 (50.28)			503 (56.65)	73 (53.51)		
Yes	232 (21.05)	130 (17.72)	102 (27.17)			196 (20.40)	36 (25.67)		
Unknown	237 (22.69)	138 (22.77)	99 (22.55)			208 (22.96)	29 (20.82)		
Hypertension, *n* (%)				*χ* ^2^ = 4.57	0.032			*χ* ^2^ = 4.88	0.027
No	119 (12.51)	83 (15.13)	36 (7.70)			109 (13.46)	10 (5.82)		
Yes	926 (87.49)	553 (84.87)	373 (92.30)			798 (86.54)	128 (94.18)		
Dyslipidemia, *n* (%)				*χ* ^2^ = 1.88	0.171			*χ* ^2^ = 0.00	0.975
No	87 (9.43)	43 (8.27)	44 (11.56)			75 (9.44)	12 (9.36)		
Yes	958 (90.57)	593 (91.73)	365 (88.44)			832 (90.56)	126 (90.64)		
CVD, *n* (%)				*χ* ^2^ = 16.96	<0.001			*χ* ^2^ = 9.31	0.002
No	452 (45.48)	313 (51.71)	139 (34.03)			409 (47.93)	43 (28.08)		
Yes	593 (54.52)	323 (48.29)	270 (65.97)			498 (52.07)	95 (71.92)		
Depression, *n* (%)				*χ* ^2^ = 0.22	0.643			*χ* ^2^ = 1.02	0.312
No	784 (73.57)	481 (74.18)	303 (72.43)			684 (74.17)	100 (69.26)		
Yes	261 (26.43)	155 (25.82)	106 (27.57)			223 (25.83)	38 (30.74)		
Family history of diabetes, *n* (%)				*χ* ^2^ = 3.73	0.054			*χ* ^2^ =2.10	0.147
No	348 (33.62)	189 (30.61)	159 (39.17)			296 (32.64)	52 (40.61)		
Yes	697 (66.38)	447 (69.39)	250 (60.83)			611 (67.36)	86 (59.39)		
BMI, *n* (%)				*χ* ^2^ = 17.72	<0.001			*χ* ^2^ = 0.76	0.384
BMI < 25 kg/m^2^	146 (11.46)	62 (7.42)	84 (18.88)			127 (11.09)	19 (14.04)		
BMI ≥ 25 kg/m^2^	899 (88.54)	574 (92.58)	325 (81.12)			780 (88.91)	119 (85.96)		
Total energy, kcal, mean (S.E)	1909.45 (50.60)	1992.20 (67.30)	1757.23 (60.22)	*t* = 2.71	0.009	1930.11 (53.49)	1762.95 (118.50)	*t* = 1.33	0.189
Carbohydrate, g, mean (S.E)	47.84 (0.67)	47.33 (1.01)	48.79 (0.83)	*t* = −1.02	0.312	47.98 (0.77)	46.89 (1.26)	*t* = 0.68	0.497
Protein, g, Mean (S.E)	16.07 (0.32)	16.07 (0.41)	16.07 (0.34)	*t* = 0.01	0.991	15.98 (0.35)	16.72 (0.56)	*t* = −1.15	0.255
Fat, g, mean (S.E)	35.48 (0.36)	35.75 (0.52)	35.00 (0.60)	*t* = 0.84	0.403	35.48 (0.42)	35.50 (1.11)	*t* = −0.02	0.986
Fiber, g, mean (S.E)	1.73 (0.06)	1.70 (0.06)	1.81 (0.13)	*t* = −0.82	0.414	1.70 (0.05)	1.96 (0.36)	*t* = −0.71	0.479
Total sugar, g, mean (S.E)	19.87 (0.47)	19.28 (0.62)	20.95 (0.96)	*t* = −1.34	0.186	20.01 (0.55)	18.85 (1.06)	*t* = 0.89	0.379
Uric acid, mg/dL, mean (S.E)	6.15 (0.08)	5.86 (0.10)	6.66 (0.12)	*t* = −5.07	<0.001	6.06 (0.09)	6.76 (0.19)	*t* = −3.32	0.002
ALT, U/L, mean (S.E)	27.01 (1.26)	27.33 (1.22)	26.43 (2.71)	*t* = 0.30	0.762	27.46 (1.38)	23.86 (1.32)	*t* = 2.10	0.041
AST, U/L, mean (S.E)	26.62 (0.81)	25.70 (0.92)	28.31 (1.44)	*t* = −1.59	0.119	26.44 (0.85)	27.91 (1.52)	*t* = −0.94	0.353
Neutrophils, 1000 cell/*u*L, mean (S.E)	4.95 (0.07)	4.97 (0.10)	4.92 (0.12)	*t* = 0.33	0.746	4.99 (0.08)	4.67 (0.14)	*t* = 1.98	0.054
Lymphocyte, 1000 cell/*u*L, mean (S.E)	2.16 (0.05)	2.27 (0.06)	1.96 (0.06)	*t* = 4.08	<0.001	2.20 (0.05)	1.88 (0.09)	*t* = 3.28	0.002
Platelet, 1000 cell/*u*L, mean (S.E)	243.49 (3.92)	246.28 (5.23)	238.35 (4.55)	*t* = 1.21	0.234	243.82 (4.31)	241.09 (7.34)	*t* = 0.32	0.747
Hemoglobin, g/dL, mean (S.E)	13.82 (0.09)	14.04 (0.12)	13.43 (0.12)	*t* = 3.80	<0.001	13.87 (0.10)	13.50 (0.18)	*t* = 1.77	0.082
Calcium, mmol/L, mean (S.E)	2.34 (0.01)	2.34 (0.01)	2.35 (0.01)	*t* = −0.26	0.798	2.34 (0.01)	2.34 (0.01)	*t* = 0.59	0.558
Potassium, mmol/L, mean (S.E)	4.19 (0.03)	4.16 (0.03)	4.24 (0.03)	*t* = −1.94	0.058	4.18 (0.03)	4.22 (0.05)	*t* = −0.71	0.483
Phosphorus, mmol/L, mean (S.E)	1.19 (0.01)	1.17 (0.01)	1.24 (0.02)	*t* = −4.11	<0.001	1.18 (0.01)	1.26 (0.03)	*t* = −2.86	0.006
Nephrotoxic drugs, *n* (%)				*χ* ^2^ = 0.11	0.743			*χ* ^2^ = 1.19	0.276
No	962 (92.33)	582 (92.54)	380 (91.95)			832 (92.02)	130 (94.54)		
Yes	83 (7.67)	54 (7.46)	29 (8.05)			75 (7.98)	8 (5.46)		
Dialysis, *n* (%)				*χ* ^2^ = 5.03	0.081			*χ* ^2^ = 0.10	0.953
No	143 (12.89)	79 (11.33)	64 (15.75)			126 (13.01)	17 (12.02)		
Yes	26 (1.60)	13 (1.46)	13 (1.86)			23 (1.63)	3 (1.39)		
Unknown	876 (85.51)	544 (87.21)	332 (82.39)			758 (85.36)	118 (86.59)		
Antidiabetic agent, *n* (%)				*χ* ^2^ = 0.06	0.813			*χ* ^2^ = 0.24	0.627
No	262 (24.80)	154 (24.54)	108 (25.27)			228 (25.05)	34 (23.01)		
Yes	783 (75.20)	482 (75.46)	301 (74.73)			679 (74.95)	104 (76.99)		
eGFR, mL/min/1.73m^2^, Mean (S.E)	81.92 (1.40)	88.30 (1.73)	69.61 (1.82)	*t* = 7.34	<0.001	83.11 (1.43)	72.84 (3.24)	*t* = 3.11	0.003
UACR, mg/g, mean (S.E)	340.69 (39.16)	295.03 (38.33)	425.58 (82.65)	*t* = −1.45	0.153	321.90 (32.78)	474.38 (187.48)	*t* = −0.82	0.416
Follow-up time, months, mean (S.E)	76.36 (3.24)	76.84 (3.83)	75.45 (4.19)	*t* = 0.29	0.772	76.05 (3.33)	78.53 (5.30)	*t* = −0.50	0.621

Abbreviations: *χ*^2^: chi-square test; BMI, body mass index; CVD, cardiovascular disease; DKD, diabetic kidney disease; eGFR, estimated glomerular filtration rate; S.E: standard error; T: *t*-test; UACR, urinary albumin-to-creatinine ratio.

**Table 2:** Association of flavonoids with all-cause and CVD mortality among DKD patients.

**Variables**	**n** ** (%)**	**All-cause mortality**		**CVD mortality**	
		**HR (95% CI)**	***P***	**HR (95% CI)**	**p**
Total flavonoids, *n* (%)					
≤ 11.74 mg/1000 kcal	342 (33.29)	Ref		Ref	
11.74–51.99 mg/1000 kcal	379 (33.36)	0.94 (0.72–1.23)	0.654^a^	0.83 (0.42–1.66)	0.596^b^
>51.99 mg/1000 kcal	324 (33.36)	0.69 (0.52–0.92)	0.013^a^	0.98 (0.58–1.66)	0.938^b^
Isoflavones, *n* (%)					
0 mg/1000 kcal	641 (62.09)	Ref		Ref	
0–0.03 mg/1000 kcal	199 (19.11)	1.05 (0.79–1.41)	0.720^a^	1.18 (0.72–1.93)	0.500^b^
>0.03 mg/1000 kcal	205 (18.80)	0.81 (0.55–1.20)	0.289^a^	0.95 (0.42–2.15)	0.898^b^
Anthocyanidins, *n* (%)					
0 mg/1000 kcal	379 (35.94)	Ref		Ref	
0–2.00 mg/1000 kcal	351 (32.81)	0.81 (0.62–1.06)	0.121^a^	0.54 (0.35–0.85)	0.008^b^
>2.00 mg/1000 kcal	315 (31.25)	0.84 (0.61–1.15)	0.270^a^	0.64 (0.40–1.02)	0.063^b^
Flavan-3-ols, *n* (%)					
≤ 4.01 mg/1000 kcal	338 (33.29)	Ref		Ref	
4.01–22.46 mg/1000 kcal	372 (32.86)	0.99 (0.72–1.38)	0.978^a^	0.73 (0.39–1.39)	0.331^b^
> 22.46 mg/1000 kcal	335 (33.85)	0.75 (0.55–1.03)	0.074^a^	0.81 (0.48–1.36)	0.419^b^
Flavanones, *n* (%)					
0 mg/1000 kcal	444 (40.52)	Ref		Ref	
0–0.75 mg/1000 kcal	291 (29.50)	1.04 (0.72–1.51)	0.820^a^	0.67 (0.39–1.14)	0.133^b^
> 0.75 mg/1000 kcal	310 (29.98)	1.21 (0.87–1.68)	0.254^a^	1.06 (0.64–1.74)	0.823^b^
Flavonols, *n* (%)					
≤ 3.46 mg/1000 kcal	323 (33.22)	Ref		Ref	
3.46–9.67 mg/1000 kcal	386 (33.45)	0.84 (0.59–1.20)	0.334^a^	1.63 (0.92–2.88)	0.094^b^
> 9.67 mg/1000 kcal	336 (33.33)	0.77 (0.57–1.04)	0.082^a^	1.38 (0.75–2.52)	0.291^b^
Flavones, *n* (%)					
≤ 0.06 mg/1000 kcal	325 (32.97)	Ref		Ref	
0.06–0.37 mg/1000 kcal	349 (33.63)	0.59 (0.39–0.88)	0.011^a^	0.60 (0.37–0.97)	0.037^b^
> 0.37 mg/1000 kcal	371 (33.40)	0.60 (0.45–0.80)	<0.001^a^	0.50 (0.28–0.87)	0.016^b^

Abbreviations: CI: confidence interval; CVD, cardiovascular disease; DKD, diabetic kidney disease; HR: hazard ratio; Ref: reference.

^a^Adjusted for age, race, education levels, marriage, physical activity, diabetic retinopathy, hypertension, CVD, overweight, uric acid, lymphocyte, platelet, hemoglobin, potassium intake, phosphorus intake, dialysis, gender, eGFR, and UACR.

^b^Adjusted for age, race, hypertension, CVD, uric acid, lymphocyte, hemoglobin, calcium intake, potassium intake, phosphorus intake, gender, eGFR, and UACR.

**Table 3:** Association between the subclasses of flavones with all-cause mortality among DKD patients.

**Variables**	**n** ** (%)**	**HR (95% CI)** ^a^	**p**
Flavones (mg/1000 kcal)			
Apigenin			
≤ 0.01	345 (33.2)	Ref	
0.01–0.04	329 (33.37)	0.77 (0.53–1.13)	0.183
> 0.04	371 (33.42)	0.65 (0.48–0.87)	0.004
Luteolin			
≤0.04	325 (33.18)	Ref	
0.04–0.26	354 (33.45)	0.69 (0.46–1.02)	0.061
> 0.26	366 (33.38)	0.68 (0.52–0.88)	0.005

Abbreviations: CI: confidence interval; CVD, cardiovascular disease; DKD, diabetic kidney disease. HR: hazard ratio; Ref: reference.

^a^Adjusted age, race, education, marriage, physical activity, diabetic retinopathy, hypertension, CVD, overweight, uric acid, lymphocyte, platelet, hemoglobin, potassium, phosphorus, dialysis, gender, eGFR, and UACR.

**Table 4:** Association between the subclasses of anthocyanidins and flavones with CVD mortality among DKD patients.

**Variables**	**N** ** (%)**	**HR (95% CI)** ^a^	**p**
Anthocyanidins(mg/1000 kcal)			
Cyanidin			
0	390 (37.6)	Ref	
0–0.48	317 (31.19)	0.58 (0.33–1.02)	0.060
> 0.48	338 (31.21)	0.75 (0.54–1.04)	0.085
Petunidin			
0	764 (71.05)	Ref	
0–0.18	133 (14.34)	0.77 (0.39–1.50)	0.433
> 0.18	148 (14.61)	0.98 (0.55–1.75)	0.956
Delphinidin			
0	698 (64.24)	Ref	
0–0.26	177 (17.4)	0.97 (0.62–1.53)	0.895
> 0.26	170 (18.37)	0.92 (0.59–1.42)	0.686
Malvidin			
0	799 (74.33)	Ref	
0–1.69	114 (12.56)	0.95 (0.55–1.65)	0.861
> 1.69	132 (13.11)	0.97 (0.59–1.60)	0.893
Pelargonidin			
0	637 (59.49)	Ref	
0–0.15	218 (20.22)	0.58 (0.35–0.95)	0.033
> 0.15	190 (20.29)	0.89 (0.49–1.60)	0.682
Peonidin			
0	559 (53.85)	Ref	
0–0.21	246 (23.14)	0.83 (0.50–1.37)	0.456
> 0.21	240 (23.01)	0.95 (0.58–1.53)	0.820
Flavones(mg/1000 kcal)			
Apigenin			
≤ 0.01	345 (33.2)	Ref	
0.01–0.04	329 (33.37)	0.58 (0.32–1.06)	0.077
> 0.04	371 (33.42)	0.70 (0.43–1.15)	0.156
Luteolin			
≤ 0.04	325 (33.18)	Ref	
0.04–0.26	354 (33.45)	0.85 (0.46–1.55)	0.578
> 0.26	366 (33.38)	0.52 (0.31–0.88)	0.017

Abbreviations: CI: confidence interval; CVD, cardiovascular disease; DKD, diabetic kidney disease; HR: hazard ratio; Ref: reference.

^a^Adjusted age, race, hypertension, CVD, uric acid, lymphocyte, hemoglobin, calcium, potassium, phosphorus, gender, eGFR, and UACR.

**Table 5:** Association of flavonoid intake with all-cause mortality in different smoking status and BMI subgroups.

**Subgroup**	**Model**			
	**HR (95% CI)**	**p**	**HR (95% CI)**	**p**
Subgroup I: smoking	No		Yes	
Flavones				
≤ 0.06	Ref		Ref	
0.06~0.37	0.68 (0.37–1.26)	0.215	0.48 (0.29–0.79)	0.004
> 0.37	0.56 (0.34–0.94)	0.029	0.62 (0.43–0.88)	0.009
Apigenin				
≤ 0.01	Ref		Ref	
0.01~0.04	0.91 (0.51–1.63)	0.755	0.71 (0.46–1.12)	0.136
> 0.04	0.68 (0.36–1.30)	0.235	0.63 (0.42–0.96)	0.032
Luteolin				
≤ 0.04	Ref		Ref	
0.04~0.26	0.69 (0.43–1.12)	0.134	0.60 (0.36–1.01)	0.054
> 0.26	0.60 (0.37–0.97)	0.039	0.72 (0.51–1.03)	0.072
Subgroup II: BMI	BMI < 25 kg/m^2^		BMI ≥ 25 kg/m^2^	
Flavones				
≤ 0.06	Ref		Ref	
0.06~0.37	0.17 (0.06–0.46)	0.001	0.64 (0.43–0.96)	0.031
> 0.37	0.21 (0.13–0.34)	<0.001	0.66 (0.47–0.92)	0.015
Apigenin				
≤ 0.01	Ref		Ref	
0.01~0.04	1.10 (0.46–2.63)	0.832	0.77 (0.50–1.18)	0.222
>0.04	0.89 (0.55–1.43)	0.613	0.63 (0.44–0.89)	0.010
Luteolin				
≤ 0.04	Ref		Ref	
0.04~0.26	0.33 (0.15–0.73)	0.009	0.74 (0.50–1.09)	0.126
> 0.26	0.26 (0.14–0.48)	<0.001	0.72 (0.52–0.99)	0.045

*Note:* Subgroup I adjusted: age, race, education, marriage, physical activity, diabetic retinopathy, hypertension, CVD, overweight, uric acid, lymphocyte, platelet, hemoglobin, potassium, phosphorus, dialysis, gender, eGFR, and UACR. Subgroup II adjusted: age, race, education, marriage, physical activity, diabetic retinopathy, hypertension, CVD, uric acid, lymphocyte, platelet, hemoglobin, potassium, phosphorus, dialysis, gender, eGFR, and UACR.

**Table 6:** Association of flavonoid intake with CVD mortality in different smoking status and BMI subgroups.

**Subgroup**	**Model**			
	**HR (95% CI)**	**p**	**HR (95% CI)**	**p**
Subgroup I: smoking	No		Yes	
Anthocyanidins				
0	Ref		Ref	
0~2.00	0.25 (0.09–0.67)	0.007	0.87 (0.43–1.77)	0.692
> 2.00	0.48 (0.23–1.02)	0.057	0.83 (0.35–1.95)	0.657
Cyanidin				
0	Ref		Ref	
0~0.48	0.23 (0.10–0.53)	< 0.001	1.05 (0.48–2.28)	0.904
> 0.48	0.54 (0.28–1.07)	0.078	0.73 (0.36–1.49)	0.384
Petunidin				
0	Ref		Ref	
0~0.18	0.64 (0.19–2.14)	0.461	0.99 (0.44–2.23)	0.980
>0.18	0.57 (0.20–1.66)	0.298	1.37 (0.52–3.61)	0.513
Delphinidin				
0	Ref		Ref	
0~0.26	0.91 (0.35–2.41)	0.854	1.13 (0.58–2.22)	0.711
> 0.26	0.62 (0.24–1.58)	0.305	1.13 (0.47–2.75)	0.779
Malvidin				
0	Ref		Ref	
0~1.69	0.77 (0.31–1.91)	0.568	1.17 (0.48–2.88)	0.725
> 1.69	0.66 (0.23–1.92)	0.440	1.26 (0.57–2.81)	0.562
Pelargonidin				
0	Ref		Ref	
0~0.15	0.50 (0.20–1.24)	0.130	0.47 (0.18–1.21)	0.114
> 0.15	0.93 (0.31–2.74)	0.892	0.77 (0.36–1.63)	0.486
Peonidin				
0	Ref		Ref	
0~0.21	0.62 (0.28–1.36)	0.226	0.78 (0.37–1.64)	0.505
> 0.21	0.71 (0.25–2.02)	0.511	0.96 (0.43–2.17)	0.922
Flavones				
≤ 0.06	Ref		Ref	
0.06~0.37	0.58 (0.25–1.36)	0.209	0.53 (0.28–0.99)	0.051
> 0.37	0.62 (0.27–1.39)	0.236	0.40 (0.21–0.75)	0.005
Apigenin				
≤ 0.01	Ref		Ref	
0.01~0.04	0.73 (0.23–2.32)	0.586	0.47 (0.23–0.94)	0.033
> 0.04	0.86 (0.32–2.28)	0.751	0.55 (0.28–1.07)	0.078
Luteolin				
≤ 0.04	Ref		Ref	
0.04~0.26	0.86 (0.39–1.93)	0.716	0.73 (0.38–1.39)	0.331
> 0.26	0.55 (0.27–1.10)	0.090	0.44 (0.22–0.89)	0.023
Subgroup II: BMI	BMI< 25 kg/m^2^		BMI ≥25 kg/m^2^	
Anthocyanidins				
0	Ref		Ref	
0~2.00	0.00 (0.00–0.12)	0.005	0.68 (0.42–1.11)	0.120
> 2.00	0.80 (0.24–2.72)	0.716	0.63 (0.38–1.05)	0.076
Cyanidin				
0	Ref		Ref	
0~0.48	0.04 (0.00–0.37)	0.006	0.65 (0.37–1.14)	0.130
> 0.48	0.26 (0.15–0.46)	<0.001	0.70 (0.44–1.12)	0.131
Petunidin				
0	Ref		Ref	
0~0.18	0.10 (0.01–1.16)	0.064	0.85 (0.43–1.68)	0.643
> 0.18	0.85 (0.33–2.20)	0.724	0.86 (0.46–1.60)	0.618
Delphinidin				
0	Ref		Ref	
0~0.26	0.20 (0.02–1.90)	0.152	1.07 (0.66–1.73)	0.774
> 0.26	1.76 (0.98–3.17)	0.057	0.75 (0.43–1.30)	0.302
Malvidin				
0	Ref		Ref	
0~1.69	0.80 (0.19–3.42)	0.753	0.82 (0.44–1.53)	0.533
> 1.69	0.21 (0.07–0.59)	0.005	0.93 (0.50–1.73)	0.822
Pelargonidin				
0	Ref		Ref	
0~0.15	0.06 (0.01–0.46)	0.009	0.58 (0.28–1.19)	0.137
> 0.15	1.53 (0.60–3.95)	0.360	0.74 (0.41–1.33)	0.309
Peonidin				
0	Ref		Ref	
0~0.21	0.14 (0.05–0.43)	0.001	0.85 (0.41–1.79)	0.671
> 0.21	1.11 (0.39–3.16)	0.838	0.89 (0.50–1.58)	0.677
Flavones				
≤ 0.06	Ref		Ref	
0.06~0.37	0.04 (0.01–0.27)	0.002	0.70 (0.42–1.18)	0.175
> 0.37	0.50 (0.17–1.50)	0.204	0.47 (0.25–0.88)	0.019
Apigenin				
≤ 0.01	Ref		Ref	
0.01~0.04	0.33 (0.02–7.26)	0.470	0.53 (0.29–0.97)	0.039
> 0.04	0.53 (0.23–1.21)	0.128	0.66 (0.38–1.14)	0.132
Luteolin				
≤ 0.04	Ref		Ref	
0.04~0.26	2.12 (0.43–10.41)	0.339	0.86 (0.49–1.49)	0.575
> 0.26	1.53 (0.26–9.10)	0.630	0.46 (0.26–0.83)	0.011

*Note:* Subgroup I/II adjusted: age, race, hypertension, CVD, uric acid, lymphocyte, hemoglobin, calcium, potassium, phosphorus, gender, eGFR, and UACR.

## Data Availability

All data from this study were publicly available (https://www.cdc.gov/nchs/nhanes).
