# Supplementary material for: Association of Dietary Flavonoids Intake With All-Cause and Cardiovascular Disease Mortality in Diabetic Kidney Disease: A Cohort Study From the NHANES Database
Source: J Diabetes Res. 2024 Nov 4;2024:8359294. doi: 10.1155/2024/8359294 (PMC11554414; doi:10.1155/2024/8359294)
Supplement: Supporting Information — Additional supporting information can be found online in the Supporting Information section. Table S1: comparison before and after imputation. [file 8359294.f1.docx]

Table S1 Comparison before and after imputation.

| Variables | Before imputation | After imputation | Statistics | *P* |
| --- | --- | --- | --- | --- |
| Education, n (%) |  |  | χ^2^=4.01 | 0.135 |
| Below high school | 420 (29.18) | 423 (29.40) |  |  |
| High school | 236 (29.58) | 236 (29.47) |  |  |
| College graduate or above | 385 (41.24) | 386 (41.13) |  |  |
| Marriage, n (%) |  |  | χ^2^=2.00 | 0.368 |
| Married | 525 (53.62) | 526 (53.64) |  |  |
| Never married | 87 (7.31) | 87 (7.30) |  |  |
| Others | 432 (39.07) | 432 (39.06) |  |  |
| Smoking, n (%) |  |  | χ^2^=1.00 | 0.318 |
| No | 496 (47.34) | 497 (47.36) |  |  |
| Yes | 548 (52.66) | 548 (52.64) |  |  |
| Family history of diabetes, n (%) |  |  | χ^2^=0.17 | 0.678 |
| No | 335 (33.75) | 348 (33.62) |  |  |
| Yes | 674 (66.25) | 697 (66.38) |  |  |
| BMI, n (%) |  |  | χ^2^=0.36 | 0.547 |
| BMI<25kg/m^2^ | 138 (11.36) | 146 (11.46) |  |  |
| BMI≥25kg/m^2^ | 876 (88.64) | 899 (88.54) |  |  |
| Uric acid, mg/dL, Mean (S.E) | 6.13 (0.09) | 6.15 (0.08) | t=-1.62 | 0.112 |
| ALT, U/L, Mean (S.E) | 27.03 (1.32) | 27.01 (1.26) | t=0.16 | 0.875 |
| AST, U/L, Mean (S.E) | 26.48 (0.84) | 26.62 (0.81) | t=-1.20 | 0.235 |
| Neutrophils, 1000 cell/uL, Mean (S.E) | 4.96 (0.08) | 4.95 (0.07) | t=1.32 | 0.195 |
| Lymphocyte, 1000 cell/uL, Mean (S.E) | 2.16 (0.05) | 2.16 (0.05) | t=0.77 | 0.444 |
| Platelet, 1000 cell/uL, Mean (S.E) | 243.64 (4.01) | 243.49 (3.92) | t=0.53 | 0.599 |
| Hemoglobin, g/dL, Mean (S.E) | 13.83 (0.09) | 13.82 (0.09) | t=1.27 | 0.211 |
| Calcium, mmol/L, Mean (S.E) | 2.34 (0.01) | 2.34 (0.01) | t=-0.43 | 0.668 |
| Potassium, mmol/L, Mean (S.E) | 4.19 (0.03) | 4.19 (0.03) | t=-0.49 | 0.625 |
| Phosphorus, mmol/L, Mean (S.E) | 1.19 (0.01) | 1.19 (0.01) | t=-1.91 | 0.063 |

T: t-test; χ^2^: chi-square test; S.E: standard error.

BMI, body mass index.

Table S2 Dietary flavonoids intake levels of DKD patients.

| Variables | Total (n=1045) | All-cause mortality | | | | CVD mortality | | | |
| --- | --- | --- | --- | --- | --- | --- | --- | --- | --- |
|  |  | No (n=636) | Yes (n=409) | Statistics | *P* | No (n=907) | Yes (n=138) | Statistics | *P* |
| Total Flavonoids, mg/1000kcal, Mean (S.E) | 114.23 (13.26) | 103.97 (13.07) | 133.11 (28.10) | t=-0.95 | 0.349 | 106.72 (11.13) | 167.50 (57.84) | t=-1.08 | 0.286 |
| Total Flavonoids, n (%) |  |  |  | χ^2^=2.55 | 0.279 |  |  | χ^2^=3.13 | 0.209 |
| ≤11.74 mg/1000kcal | 342 (33.29) | 215 (35.12) | 127 (29.91) |  |  | 301 (33.95) | 41 (28.59) |  |  |
| 11.74 - 51.99 mg/1000kcal | 379 (33.36) | 227 (31.50) | 152 (36.79) |  |  | 336 (33.75) | 43 (30.56) |  |  |
| > 51.99 mg/1000kcal | 324 (33.36) | 194 (33.39) | 130 (33.30) |  |  | 270 (32.30) | 54 (40.85) |  |  |
| Isoflavones, mg/1000kcal, Mean (S.E) | 0.58 (0.16) | 0.51 (0.15) | 0.71 (0.40) | t=-0.44 | 0.659 | 0.55 (0.18) | 0.80 (0.59) | t=-0.38 | 0.705 |
| Isoflavones, n (%) |  |  |  | χ^2^=0.95 | 0.623 |  |  | χ^2^=0.29 | 0.867 |
| 0 mg/1000kcal | 641 (62.09) | 371 (60.78) | 270 (64.49) |  |  | 551 (61.93) | 90 (63.18) |  |  |
| 0 - 0.03 mg/1000kcal | 199 (19.11) | 130 (19.44) | 69 (18.53) |  |  | 175 (18.98) | 24 (20.06) |  |  |
| >0.03 mg/1000kcal | 205 (18.80) | 135 (19.78) | 70 (16.99) |  |  | 181 (19.09) | 24 (16.76) |  |  |
| Anthocyanidins, mg/1000kcal, Mean (S.E) | 7.13 (1.09) | 7.06 (1.28) | 7.27 (1.86) | t=-0.09 | 0.925 | 7.14 (1.13) | 7.10 (2.87) | t=0.01 | 0.989 |
| Anthocyanidins, n (%) |  |  |  | χ^2^=1.31 | 0.520 |  |  | χ^2^=6.55 | 0.038 |
| 0 mg/1000kcal | 379 (35.94) | 221 (34.97) | 158 (37.72) |  |  | 321 (34.51) | 58 (46.03) |  |  |
| 0 - 2.00 mg/1000kcal | 351 (32.81) | 226 (34.12) | 125 (30.41) |  |  | 311 (33.63) | 40 (27.00) |  |  |
| >2.00 mg/1000kcal | 315 (31.25) | 189 (30.91) | 126 (31.87) |  |  | 275 (31.85) | 40 (26.97) |  |  |
| Flavan-3-ols, mg/1000kcal, Mean (S.E) | 126.44 (17.36) | 114.81 (17.14) | 147.82 (37.02) | t=-0.81 | 0.421 | 116.62 (14.49) | 196.06 (78.39) | t=-1.04 | 0.306 |
| Flavan-3-ols, n (%) |  |  |  | χ^2^=1.08 | 0.584 |  |  | χ^2^=0.99 | 0.611 |
| ≤4.01 mg/1000kcal | 338 (33.29) | 202 (33.28) | 136 (33.31) |  |  | 293 (32.84) | 45 (36.50) |  |  |
| 4.01 - 22.46 mg/1000kcal | 372 (32.86) | 225 (31.53) | 147 (35.30) |  |  | 330 (33.38) | 42 (29.15) |  |  |
| > 22.46 mg/1000kcal | 335 (33.85) | 209 (35.19) | 126 (31.39) |  |  | 284 (33.78) | 51 (34.35) |  |  |
| Flavanones, mg/1000kcal, Mean (S.E) | 7.19 (0.68) | 5.68 (0.73) | 9.96 (1.16) | t=-3.58 | <0.001 | 6.91 (0.73) | 9.18 (1.38) | t=-1.50 | 0.140 |
| Flavanones, n (%) |  |  |  | χ^2^=8.36 | 0.015 |  |  | χ^2^=7.46 | 0.024 |
| 0 mg/1000kcal | 444 (40.52) | 280 (43.59) | 164 (34.88) |  |  | 383 (40.33) | 61 (41.83) |  |  |
| 0 - 0.75 mg/1000kcal | 291 (29.50) | 186 (30.43) | 105 (27.81) |  |  | 260 (30.88) | 31 (19.75) |  |  |
| > 0.75 mg/1000kcal | 310 (29.98) | 170 (25.99) | 140 (37.31) |  |  | 264 (28.79) | 46 (38.42) |  |  |
| Flavonols, mg/1000kcal, Mean (S.E) | 9.48 (0.57) | 8.81 (0.56) | 10.72 (1.22) | t=-1.43 | 0.158 | 8.99 (0.50) | 12.94 (2.33) | t=-1.73 | 0.091 |
| Flavonols, n (%) |  |  |  | χ^2^=8.53 | 0.014 |  |  | χ^2^=11.07 | 0.004 |
| ≤3.46 mg/1000kcal | 323 (33.22) | 204 (36.22) | 119 (27.70) |  |  | 291 (35.04) | 32 (20.28) |  |  |
| 3.46 - 9.67 mg/1000kcal | 386 (33.45) | 230 (30.25) | 156 (39.34) |  |  | 331 (31.99) | 55 (43.87) |  |  |
| > 9.67 mg/1000kcal | 336 (33.33) | 202 (33.53) | 134 (32.95) |  |  | 285 (32.97) | 51 (35.85) |  |  |
| Flavones, mg/1000kcal, Mean (S.E) | 0.43 (0.04) | 0.44 (0.06) | 0.43 (0.04) | t=0.14 | 0.888 | 0.44 (0.04) | 0.39 (0.08) | t=0.52 | 0.609 |
| Flavones, n (%) |  |  |  | χ^2^=0.44 | 0.804 |  |  | χ^2^=2.45 | 0.294 |
| ≤0.06 mg/1000kcal | 325 (32.97) | 191 (32.60) | 134 (33.67) |  |  | 276 (31.92) | 49 (40.43) |  |  |
| 0.06 - 0.37 mg/1000kcal | 349 (33.63) | 214 (34.59) | 135 (31.86) |  |  | 303 (34.00) | 46 (30.97) |  |  |
| > 0.37 mg/1000kcal | 371 (33.40) | 231 (32.81) | 140 (34.47) |  |  | 328 (34.07) | 43 (28.59) |  |  |

T: t-test; χ^2^: chi-square test; S.E: standard error;

DKD, diabetic kidney disease; CVD, cardiovascular disease.
